# Silver Nanoparticles: Multifunctional Tool in Environmental Water Remediation

**DOI:** 10.3390/ma17091939

**Published:** 2024-04-23

**Authors:** Pamela Nair Silva-Holguín, Jesús Alberto Garibay-Alvarado, Simón Yobanny Reyes-López

**Affiliations:** Laboratorio de Materiales Híbridos Nanoestructurados, Departamento de Ciencias Químico-Biológicas, Instituto de Ciencias Biomédicas, Universidad Autónoma de Ciudad Juárez, Envolvente del PRONAF y Estocolmo s/n, Ciudad Juárez 32300, Mexico; al231304@alumnos.uacj.mx (P.N.S.-H.);

**Keywords:** silver nanoparticles, antibacterial effect, surface modification, colorimetric detection, enhanced spectroscopy, plasmonic photocatalysts

## Abstract

Water pollution is a worldwide environmental and health problem that requires the development of sustainable, efficient, and accessible technologies. Nanotechnology is a very attractive alternative in environmental remediation processes due to the multiple properties that are conferred on a material when it is at the nanometric scale. This present review focuses on the understanding of the structure–physicochemical properties–performance relationships of silver nanoparticles, with the objective of guiding the selection of physicochemical properties that promote greater performance and are key factors in their use as antibacterial agents, surface modifiers, colorimetric sensors, signal amplifiers, and plasmonic photocatalysts. Silver nanoparticles with a size of less than 10 nm, morphology with a high percentage of reactive facets {111}, and positive surface charge improve the interaction of the nanoparticles with bacterial cells and induce a greater antibacterial effect. Adsorbent materials functionalized with an optimal concentration of silver nanoparticles increase their contact area and enhance adsorbent capacity. The use of stabilizing agents in silver nanoparticles promotes selective adsorption of contaminants by modifying the surface charge and type of active sites in an adsorbent material, in addition to inducing selective complexation and providing stability in their use as colorimetric sensors. Silver nanoparticles with complex morphologies allow the formation of hot spots or chemical or electromagnetic bonds between substrate and analyte, promoting a greater amplification factor. Controlled doping with nanoparticles in photocatalytic materials produces improvements in their electronic structural properties, promotes changes in charge transfer and bandgap, and improves and expands their photocatalytic properties. Silver nanoparticles have potential use as a tool in water remediation, where by selecting appropriate physicochemical properties for each application, their performance and efficiency are improved.

## 1. Introduction

The introduction of foreign agents of a chemical, physical, or biological nature to a certain environment causes disturbance to that environment and can induce harmful effects because of pollution [1]. The magnitude of the negative effects on the environment will depend on the type and quantity of the contaminant. Climate change, inefficient water management, and the increase in anthropogenic activities have caused greater pollution and a reduction in available clean water, which is why there is currently a serious global environmental challenge to reduce pollution [2].

Worldwide, it is estimated that 80% of wastewater is discharged into the environment without any treatment or without being reused. The discharge of untreated or inadequately treated wastewater presents harmful effects on human health, harmful environmental effects, and economic repercussions [3]. Wastewater treatment is related to the economy of the country in question; high-income countries treat about 70% of their wastewater, while low-income countries only treat 8% [4]. Preventing the release of various contaminants into the environment is an ecological and health need, which is why treatment processes and/or efficient materials are required for their elimination in trace concentrations. The application of science and technology helps the economic development of any country [2].

The main objective in the development of new technologies for water remediation is to improve the efficiency of treatment and the use of appropriate and accessible technologies that respect the limitations of low-income countries, which implies the development of technologies with lower acquisition, installation, operation, and maintenance costs and that achieve or improve the efficiency of conventional treatments. In addition, they must be sustainable and with little or no secondary pollution [3,5].

Nanotechnology through control over material size, morphology, and chemical structure offers novel materials that could provide some water treatment systems with exceptional properties that improve and enhance the efficiency of the treatment and expand its applications [6,7,8]. Nanotechnology is based on the study and understanding of the synthesis, control, manipulation, and design of matter at a nanometric scale (less than 100 nm) in any of the dimensions of the material to obtain materials with functions and properties different from their micrometric materials due to its ratio between total surface area and mass. As the size of a material decreases, the surface area increases, which means an increase in the number of atoms exposed on the surface of the material in relation to the total mass of the solid [9]. The environmental applications of silver nanoparticles search to remedy many environmental problems, which is why this manuscript touches on important aspects in that area.

Silver nanoparticles (AgNps) are frequently used due to their properties such as excellent electrical conductivity, chemical stability, antibacterial properties, and optical properties, and their synthesis process is simple and profitable [10,11]. Their applications for environmental remediation are as follows: as antibacterial agents with enhanced effects at low concentrations against Gram-negative and positive bacteria [12]; as surface modifiers in adsorbent materials, where with the functionalization of the material they increase the textural properties, the surface charge and number of active sites are modified, which leads to an increase in the adsorbent capacity of the material, in addition to promoting selective adsorption, such as plasmonic colorimetric sensors for the detection of heavy metals through the perturbation of the plasmon of surface resonance (SPR) and a visual detection with the change in solution color [13]; as signal amplifiers in infrared spectroscopy (SEIRAS) and Raman (SERS) at concentrations lower than the detection limit of the instrument measurement [14]; and as plasmonic photocatalysts for the degradation of organic contaminants [15].

Silver nanoparticles have potential use as a tool in water remediation, covering aspects such as the detection, removal, and elimination of contaminants and microorganisms. However, a better understanding of the structure–physicochemical properties–performance relationships is required for the selection of the optimal physicochemical properties of silver nanoparticles for each application. This present review focuses on the understanding of the structure–physicochemical properties–performance relationships of silver nanoparticles, with the objective of guiding the selection of the physicochemical properties that silver nanoparticles must possess to promote greater performance and efficiency in their use as antibacterial agents, surface modifiers in the adsorption process, colorimetric sensors, signal amplifiers, and plasmonic photocatalysts.

### 1.1. Silver Nanoparticles as Antibacterial Agents

**Figure 1:**
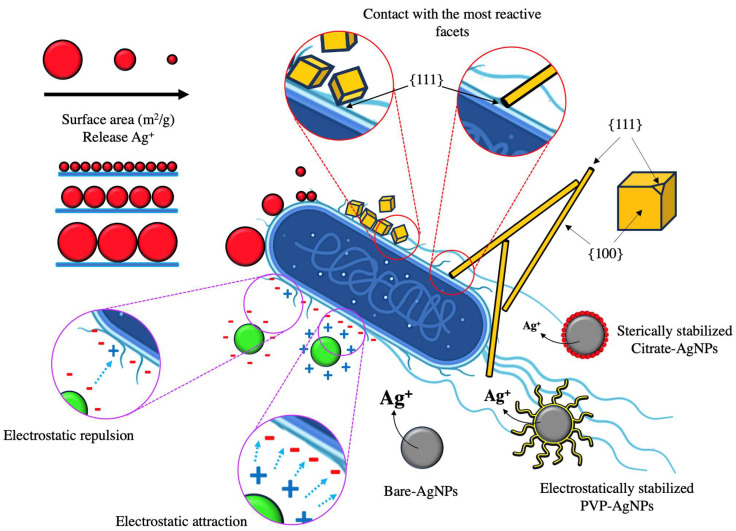
Scheme of the interaction of silver nanoparticles with a bacterial cell. Color and shape represent the different physicochemical properties of silver nanoparticles.

The antibacterial capacity of silver nanoparticles is well reported; however, there are different factors that influence the antimicrobial performance. The size, shape, crystallinity, surface charge, and the use of stabilizing agents in the nanoparticles are determining factors in the antibacterial capacity. The size of the nanoparticle is related to its surface area and the magnitude of interaction with the bacteria, in addition to its ability to release Ag^+^. Several studies have proven a strong relationship between the size and the antibacterial capacity of silver nanoparticles. For example, Wu et al., 2018 [16] and Agnohotri et al., 2014 [17] synthesized citrate-stabilized spherical silver nanoparticles with varied sizes as shown in Table 1. Both studies observed an increase in the zone of inhibition and a decrease in the minimum inhibitory concentration (MIC) by decreasing the size of silver nanoparticles. Nanoparticles with smaller sizes exhibit greater antibacterial capacity due to a greater reactivity and a greater release of silver ions. Zhang et al., 2011 [18] synthesized silver nanoparticles with different sizes and determined the release of silver ions for each size; nanoparticles of 20, 40, and 80 nm presented an Ag^+^ release of 114.4, 62.4, and 31.2 μg/L, respectively. Similarly, Sotiriou and Pratsinis, 2010 [19] determined the surface area and release of silver ions as a function of the size of the nanoparticles contained in an inert silica matrix. When synthesizing the xAg/SiO_2_ composite, they observed an increase in the size of the nanoparticles by increasing the concentration of silver in the composite. The size of the nanoparticles modified the surface area of the material, with a smaller area to larger size nanoparticles, and as the surface area decreased, the fraction of Ag^+^ ions released decreased (R^2^ = 0.96). Figure 1 shows a scheme of the interaction of silver nanoparticles with the bacterial cell when the nanoparticle has different physicochemical properties.

The shape of the nanoparticle is also related to the surface area and the release of silver ions. For example, Helmlinger et al., 2016 [20] synthesized NpsAg with different morphologies and observed a strong correlation between the specific surface area and the dissolution of the nanoparticles. The surface area estimated by geometric calculations for nanoparticles with morphology of platelets, spheres, rods, and cubes was 0.234, 0.100, 0.040, and 0.038 surface area/volume per particle/nm^2^, respectively. The dissolution rate followed the same trend as the surface area, obtaining percentage solution values by weight of Ag of 68.1% platelets, 54.3% spheres, 37.1% rods, and 30.1% cubes. Sotiriou and Pratsinis, 2010 [19] and Helmlinger et al., 2016 [20] showed a correlation between the release of silver ions and the antibacterial effect. The size of the nanoparticle is an important factor because it determines the surface area and release of Ag^+^ ions. According to the above, it is deduced that (1) by decreasing the size of the nanoparticle, the surface area increases; (2) by increasing the surface area, the release of Ag^+^ ions increase; and (3) the greater the release of ions of Ag^+^, there is greater antibacterial capacity. The morphology of the nanoparticles indicates their reactivity through the facets present in the structure. For example, Pal et al., 2007 [21] synthesized silver nanoparticles with different morphologies and suggested a relationship with the active facets present on the surface of each nanoparticle with the inhibitory capacity against *E. coli*. Triangular nanoplate nanoparticles presented greater bacterial inhibition because the upper basal plane shows facets {111} compared to spherical nanoparticles and rods that present a lower percentage of facets {111}, because spherical nanoparticles show facets {100} and a small percentage of {111}, while rod-shaped nanoparticles show {100} facets on their side surfaces, and {111} facets only at the ends. The reactivity of silver is favored by the presence of {111} facets, which have high atomic density. Wiley et al., 2005 [22] synthesized silver nanoparticles of different morphology using PVP as a stabilizing agent. They mention that the stabilizing agent PVP interacts more strongly with the {100} facets compared to the {111} ends for silver nanoparticles with cubic and rod morphology, so that a preferential adsorption occurs in the formation of the nanoparticles of the stabilizing agents (PVP) on the {100} facet, which stabilizes the {100} facet and prevents its growth; on the contrary, the {111} facet remains reactive and has a higher growth rate.

Morones et al., 2005 [23] synthesized silver nanoparticles of 21 ± 18 nm with cuboctahedral and icosahedral and multiple-twinned decahedral morphology with a mainly {111} facet surface. By observing the interaction of silver nanoparticles with the *E. coli* bacteria using STEM, they determined the presence of a 5 ± 2 nm silver nanoparticle on the surface of the bacterial membrane and demonstrated a direct interaction between the {111} facets of the nanoparticles and the bacterial surface, which indicates that the bond strength between the nanoparticles and the bacterial surface will depend on the interaction surface.

Hong et al., 2016 [24] synthesized nanocubes, nanospheres, and nanowires and observed that nanoparticles with a greater specific surface area present a more effective interaction and closer contacts with the bacteria, such as nanocubes and nanowires, exhibiting strong antibacterial activity, while nanoparticles with a smaller specific area show a weaker and inefficient interaction like nanowires, which translates into a lower antibacterial capacity. The silver nanocubes were those with the greatest antibacterial capacity, while the nanowires presented the weakest antibacterial activity. Morones et al., 2005 [23]. determined that only nanoparticles that interact with the membrane can enter the bacteria.

The type of morphology of the nanoparticle is a key factor in its reactivity and interaction–contact with the bacteria. The use of nanoparticles with a high percentage of reactive facets such as {111} allows greater reactivity and greater release of Ag^+^ ions as it is more exposed. At a higher percentage of facet {111}, there is a greater probability of contact of that facet with the bacteria, which translates into an effective interaction. The morphology also determines the surface area; having a greater surface area means a greater contact area between the nanoparticle and the bacteria, which translates into a greater antibacterial effect.

The surface charge of silver nanoparticles is a limiting factor in the interaction and adhesion with the bacterial surface. Bacteria have a negative surface charge due to the carboxyl, phosphate, and amino functional groups on their surface. For example, *E. coli* and *P. aeruginosa* when found in a medium with 50 mM phosphate buffer and a pH of 7.0 show a surface charge of −28.5 ± 2.9 mV and −20.6 ± 1.8 mV, respectively [25]. Electrostatic attractions are necessary for efficient interaction and greater inhibition. Abbaszadegan et al., 2015 [26] and Ivask et al., 2014 [27] obtained silver nanoparticles with different surface charges and observed a strong inhibitory effect by positively charged nanoparticles compared to negatively charged nanoparticles.

Positively charged nanoparticles have a greater antibacterial effect due to the existence of electrostatic interactions between the positively charged nanoparticle and the negatively charged bacterial cell, which allows greater interaction. On the other hand, when negatively charged nanoparticles are used, an electrostatic barrier is formed with a high degree of repulsion, which limits the interaction of the nanoparticles with the bacterial surface, resulting in a low antibacterial capacity. However, the nanoparticles with negative charge present weak antibacterial activity due to binding to positively charged residues of integral membrane proteins [17].

Badawy et al., 2010 [28] synthesized silver nanoparticles of spherical shape and size of 10 nm with different stabilizing agents and obtained variations in the surface charge of the nanoparticles, showing a direct correlation between the antibacterial capacity and the surface charge of the nanoparticles. Nanoparticles stabilized with citrate obtained a surface charge of −38 mV, while stabilized branched polyethyleneimine (BPEI) obtained a charge of +40 mV. The bacterial inhibition results against *Bacillus* sp. showed a marked difference, where BPEI-stabilized nanoparticles showed strong inhibitory capacity compared to citrate-stabilized nanoparticles.

The negative surface charge in the nanoparticles favors the interaction with bacteria through electrostatic attractions, and because the bactericidal character responds to a contact mechanism, the inhibitory capacity is favored. Obtaining nanoparticles with a certain surface charge is achieved using various stabilizing agents.

The use of stabilizing agents, in addition to modifying the surface of the nanoparticle, affects the release of Ag^+^ ions. Miesen et al., 2020 [29] synthesized spherical silver nanoparticles and used citrate (Cit) and sodium oleate-phosphatidylcholine (SOA-PC) as stabilizing agents when determining the release of silver ions. They observed that the Ag-Cit nanoparticles showed a release of 92.9 ± 8.6 μg/L, while the Ag-SOA-PC nanoparticles released 1228.2 ± 87.7 μg/L. The difference in the release of silver ions was attributed to the type of interaction between the nanoparticle and the stabilizing agents, which can be electrostatic repulsion, steric repulsion, or both.

Li et al., 2013 [30] determined the release of silver ions from unstabilized (naked) silver nanoparticles and with the stabilizers citrate and PVP. The nanoparticles without stabilizer presented an Ag^+^ release of 15.89 μg/L, with PVP 10.9 μg/L and with citrate 6.52 μg/L. The use of stabilizing agents on silver nanoparticles produces a protective effect, providing a physical barrier; however, the coating is generally not an impermeable layer, but rather presents discontinuity, which allows the interaction of the medium with the nanoparticle. Table 1 summarizes some of the physicochemical properties of silver nanoparticles and antibacterial capacity from the literature.

**Table 1:** Physicochemical properties of silver nanoparticles and their antibacterial capacity.

Ref.	Form	Size (nm)	SPR (nm)	Stabilizer	Zeta Potential (mV)	ZoI (mm)	MIC (μg/mL)
[16]	Spherical	2.3 ± 0.5	393	Citrate		19.5	7.8
		12.5 ± 2.2	404			16.2	15.6
		32.4 ± 6.5	423			11	62.5
[31]	Spherical	40	430			2.22 ± 0.55	
	Hexagonal	40	416/650			2.45 ± 0.24	
	Triangular	40	344/600			0.08 ± 0.14	
[24]	Spheres	60 ± 15	430				75 ± 2.6
	Cubes	55 ± 10	460				37.5 ± 5.3
	Wires	60/2–4 μm	350/390				100 ± 5.3
[32]	Spherical	44	445	PEG		3	
		39	438	EDTA		4	
		35	431	PVP		6	
		31	425	PVA		7	
[33] ^1^	Spherical	10 ± 5					1.0
		30 ± 5					2.4
		60 ± 5					7.2
		90 ± 5					11.5
[34]	Spherical	40–50	430	Citrate			190
	Rod-shaped	20/90	437/346				430
[17]	Spherical	5 ± 0.7	393	Citrate	−22.8 ± 0.8	12.4	20
		7 ± 1.3	394		−27.3 ± 1.2	11.2	20
		10 ± 2.0	398		−30.2 ± 1.2	11.1	30
		15 ± 2.3	401		−34.0 ± 2.0	6	30
		20 ± 2.5	406		−35.1 ± 0.9		40
		30 ± 5.1	411		−33.7 ± 2.0		50
		50 ± 7.6	420		−41.8 ± 1.3		80
		63 ± 7.4	429		−48.5 ± 0.9		90
		85 ± 8.2	449		−52.4 ± 3.4		90
		100 ± 11.3	462		−53.1 ± 1.8		110
[26] ^3^	Spherical	7.5	400	Sodium borohydride	−38.0		9.7 × 10^−8^
		10.1	400	Rice starch	0.0		4 × 10^−9^
		9.0	400	1-dodecyl-3-methylimidazolium	+50.0		5.7 × 10^−12^
[27] ^4^	Spherical	9.1 ± 4.2	390	Citrato	−26.3 ± 2.6		6.4
		19.1 ± 6.0	404	Citrato	−33.8 ± 2.2		15.7
		43.5 ± 12	412	Citrato	−26.9 ± 1.8		40.9
		17.9 ± 7.0	402	PVP	−10.7 ± 1.8		5.5
		23.3 ± 15	420	BPEI	+33.3 ± 1.5		2.2
[35]	Spherical	30–80	426	Tri-sodium citrate		0.9 ± 0.15	
	Triangular	150	392/789	PVP		1.4 ± 0.2	
	Spherical	25–70	403	Sodium borohydride		1.1 ± 0.35	
	Spherical	15–50	397	PVP		1.5 ± 0.3	
	Spherical	30–200	504/678/735	PVP		0.7 ± 0.3	
[20] ^2^	Spheres	40–70		PVP	−6		≥12.5–25
	Spheres	120–180			−3		≥25
	Platelets	20–60			−7		≥25
	cubes	140–180			−11		≥25–50

^1^ Use *Vibrio natriegens* bacteria, ^2^ use *S. aureus* bacteria, ^3^ MIC in Mol/L, ^4^ MIC in IC50 (mg Ag/L), SPR = surface plasmon resonance, ZoI = inhibition zone.

### 1.2. Silver Nanoparticles as Surface Modifiers in Adsorption Processes

The use of nanometric adsorbent materials in adsorption processes has potential use in environmental remediation due to their reactivity, number of active sites, and high surface area [36]. Silver nanoparticles have been used to remove metal ions in aqueous media and removal percentages greater than 90% have been obtained [37,38]. However, the use of only nanoparticles in adsorption processes is limited by the recovery of the nanoparticles after treatment because the use of additional technologies is necessary. Therefore, various adsorbent materials have been functionalized with silver nanoparticles to improve the adsorbent capacity and material handling.

The physicochemical properties of a material’s surface, such as specific surface area, charge, and functional groups, play a critical role in the adsorption capacity [39]. For example, the functionalization of adsorbent materials with silver nanoparticles increases the adsorbent capacity due to the modification of the surface of the material; however, the modification depends on the material to be functionalized and the properties of the nanoparticles. Different variations can be obtained in the physicochemical properties of the adsorbent material, as seen in Table 2. 

The functionalization with NpsAg produces a variation in the textural properties (specific surface area, pore size, and pore volume) of the adsorbent material, and the increase or decrease in the textural properties will depend on the concentration used for the functionalization. For example, Kaur et al., 2021 [40] showed an increase in textural properties when functionalizing chitosan-Polyvinyl alcohol (Ch/PVA) hydrogel with a concentration of 5 mM of NpsAg. A similar study by Jing et al., 2022 [41] used 1, 3, and 5% NpsAg to functionalize bamboo-based cellulose carbon aerogel and observed an increase in the surface area with increasing silver concentration; however, the pore volume and the pore diameter showed a decreasing trend because these pores were occupied by the nanoparticles. For both studies, the adsorbent capacity increased with increasing NpsAg concentration. Other studies showed a decrease in textural properties with functionalization with NpsAg. For example, Yang et al., 2022 [42] used 0.05, 0.5 and 1 M of NpsAg to functionalize spherical activated carbon and Chang et al., 2020 [43] used 0.001, 0.01, and 0.1 M to functionalize activated carbon. In both cases, there was a reduction in the textural properties as the concentration of NpsAg increased, which attributed to the aggregation of the NpsAg and the blocking of pores due to the formation of silver aggregates, reducing the proportion of surface of the activated carbon and resulting in a decrease in the adsorbent capacity as the concentration of NpsAg increased. For all the cases mentioned, the adsorbent capacity increased with respect to the unfunctionalized material; however, among the functionalized samples, there is great variability between the increase or decrease in the adsorbent capacity with respect to the increase in the concentration of NpsAg used.

The adsorbent capacity increases with functionalization at an optimal concentration of silver nanoparticles; however, if this optimal concentration is exceeded, a decrease in the adsorbent capacity occurs, as shown by Trinh et al., 2020 [44], Van et al., 2019 [45], and Chang et al., 2020 [43], due to the saturation of the material and blocking of the adsorbent binding sites, decreasing its surface area. Therefore, the interaction between the adsorbent and the adsorbent is reduced. An indication of the optimal functionalization concentration could be the surface area because an increase in the surface area increases the adsorbent capacity; however, with a decrease in the surface area, the material becomes saturated and its adsorption decreases.

**Table 2:** Adsorbent materials functionalized with silver nanoparticles and their physicochemical properties.

Ref.	Adsorbent Material	[AgNps]	Nps Size (nm)	SBET (m^2^/g)	Dpore (nm)	Vpore (cm^3^/g)	pH_PZC_	Adsorbent Capacity (mg/g)	Adsorbate
[41]	Bamboo-Based Cellulose Carbon Aerogel (BCA)			324.99	4.92	0.40		7.953	Formaldehyde (HCHO)
	Ag/BCA	1%		329.97	4.25	0.35		21.56	
		3%		359.29	3.24	0.35		23.56	
		5%	25.42	394.20	3.54	0.29		26.75	
[46]	PDMAEMA-*g*-PET						5.3	0.274	As (III)
	Ag@PDMAEMA-*g*-PET		17.7 ± 3.5				6.7	0.357	
[47]	Sunflower husk biochar (BC)			7.02	3.54	0.004	6.1	6.83	Tetracycline (TC)
	BCA	500 mg/L	46	0.10	17.68	0.001	5.8	9.55	
[44]	Tea activated carbon (TAC)			322		0.0032	6.15	7.38	Phosphate
	AgNps-TAC	3.0%		349		0.0036	6.52	9.87	
		6.0%						9.40	
		9.0%						9.38	
[45]	Activated carbon (AC)			691.64		0.062	4.13	38.89	Methylene blue
	AgNPs-AC	0.5%		705.32		0.065	4.91	84.81	
		1.5%						61.11	
[48]	Activated carbon (AC)			691.64		0.062	4.13	7.61	Chromium (Cr (VI))
	AgNPs-AC	2%		701.65		0.061	5.09	10.33	
[42]	Spherical activated carbon (SAC)			1077.84	2.14	0.58		30.28	Dipropyl sulfide
	Ag-SAC	0.05		998.08	2.14	0.53			
		0.5		968.91	2.13	0.52		34.34	
		1 M	20–30	945.17	2.12	0.50		≈32.50	
[49]	Graphene oxide (GO)			1060.34				469.48	Methylene blue
	GO/Ag	0.01 M	25 ± 3	1328.55			<2	588.23	
[50]	Reduced graphene oxide hydrogel (rGH)			27.28	1.98	0.069		75.67	Methylene blue
	Ag/rGH	0.01 M		163.72	1.74	0.237		100.76	
[40]	Chitosan-Polyvinyl alcohol (Ch/PVA) hydrogel			6.57	1.98	0.011	11.02	20.75	Chloroacetamide herbicide butachlor
	Ch/PVA-Ag nanocomposite hydrogels	5 mM	5–20	8.316	2.33	0.016	9.77	23.81	
[43]	Activated carbon (UAC)			667.91	2.13	0.36		0.040	Formaldehyde
	AgNO_3_-AC	0.001 M	10–80	1144.88	2.34	0.66		0.468	
		0.01 M		1117.54	2.29	0.65		0.287	
		0.1 M		1007.67	2.16	0.54		0.203	

Another important factor is the pH of the medium where the adsorption process takes place. The pH affects the surface charge of the adsorbent material, as well as the species of the adsorbate present. An important property in adsorbent materials is the pH of point of zero charge (pH_PZC_), corresponding to the pH where the positive and negative charges on the surface of a material are equal and will counteract each other, thus presenting zero net charge. Therefore, at pH > pH_PZC_, the material will present a negative charge due to the deprotonation of the functional groups and at pH < pH_PZC_, the material will present a positive charge due to the protonation of the functional groups. Functionalization with silver nanoparticles modifies the pH_PZC_ of the adsorbent materials. The type of displacement depends on the properties of the nanoparticle, as well as the use of stabilizing agents. Nguyen et al., 2019 [48] synthesized activated carbon functionalized with NpsAg for the adsorption of Cr (VI). The species distribution diagram of Cr (VI) shows that at pH < 6, the dominant species are HCrO_4_^−^ and Cr_2_O_7_^2−^, while at pH > 6, the dominant species is CrO_4_^2−^. The functionalization shifted the pH_PZC_ of the activated carbon from 4.13 to 5.09. Therefore, at pH < pH_PZC_ (5.09), the material has a positive surface charge so the electrostatic attraction of the HCrO_4_^−^ and Cr_2_O_7_^2−^ species and the material is favored and results in greater adsorption of chromium (pH 4 = 13.50 mg/g), while at pH > pH_PZC_, the material has a negative surface charge, so there are repulsive forces between the material and CrO_4_^2−^, which results in a decreased adsorbent capacity (pH 10 ≈ 9.0 mg/g). Parmanbek et al., 2022 [46] used NpsAg-functionalized poly(2-(dimethylamino)ethyl methacrylate) (PDMAEMA-g-TeMs) grafted films for As (III) adsorption. PDMAEMA-g-TeMs has a pH_PZC_ of 5.3 and with functionalization it moved to 6.7. At pH 6, where the unfunctionalized material has a positive charge (pH_PZC_ = 5.3), it presents a removal percentage of approximately 34, while the functionalized material has a positive charge (pH_PZC_ = 6.7) with a removal percentage of approximately 39%. For the case of the adsorption of an anion, the displacement of pH_PZC_ to a higher pH is beneficial because it makes the surface of the material more positive and electrostatic interactions are favored, which is reflected in the adsorbent capacity.

To promote the electrostatic attractions between the adsorbent and adsorbate and increase the adsorbent capacity of anionic species, it is necessary to move the pH_PZC_ to basic pH. On the contrary, if what is wanted is to adsorb a cationic species, it is necessary to move the pH_PZC_ to pH acid. The use of a stabilizing agent in the silver nanoparticles can indicate the direction of pH_PZC_ displacement. Tomczyk and Szewczuk-Karpisz, 2022 [47] functionalized sunflower husk biochar with NpsAg stabilized with PVP and obtained a minimum pH_PZC_ displacement of 6.1 to 5.8. Furthermore, with respect to the nonfunctionalized material, the surface charge of the functionalized material decreased in charge density toward the neutral. Silver nanoparticles stabilized with PVP present a surface charge close to neutrality, because polyvinylpyrrolidone (PVP) is an uncharged polymer and it does not undergo protonation or deprotonation depending on the pH; therefore, it is inert to changes in pH [28]. Within the synthesis of nanoparticles, different stabilizing or coating agents are used to provide stability to the particle and prevent the aggregation of the nanoparticles through electrostatic repulsion, steric repulsion, or both [51,52].

In addition to stabilizing properties, capping agents can also alter the surface chemistry of nanoparticles [53]. Depending on the functional groups present in the stabilizing agent, pH_PZC_ can be moved toward basic or acidic. If it presents acidic functional groups, the material will obtain an acidic character with a negative charge and a displacement of the pH_PZC_ toward the acidic, on the other hand, if it presents basic functional groups, the material will obtain a basic character with a positive charge and a displacement of the pH_PZC_ to the basic. When the stabilizing agent has acidic and basic functional groups, it will have an amphoteric behavior with an intermediate pH_PZC_ and uncharged stabilizing agents will be inert to pH variations. Silver nanoparticles stabilized with citrate have a zeta potential of −40 mV at pH 6.9 and an acidic character, while using polyethyleneimine as a stabilizing agent a zeta potential of +8.9 mV at pH 10.5 and a basic character is obtained [28]. Consequently, the variations in the surface charge of the different types of nanoparticles are due to the structure and number of functional groups of each stabilizing agent.

**Figure 2:**
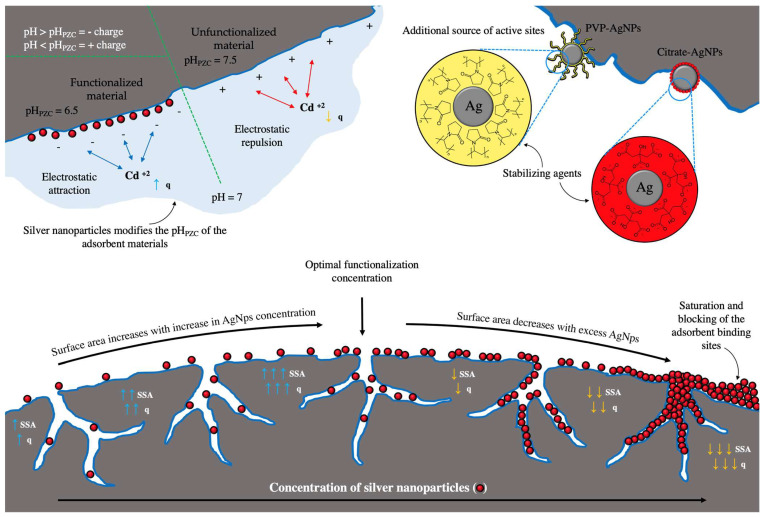
Scheme of functionalization of adsorbent material with AgNPs showing the modification of the specific surface area (SSA), active sites, surface charge, and adsorption capacity (q).

Another important aspect in the functionalization with silver nanoparticles is the contribution of new active sites to the adsorbent material. Tomczyk and Szewczuk-Karpisz, 2022 [47] observed an increase in acidic and basic sites from 2.90 and 3.20 mmol/g to 4.00 and 5.20 mmol/g with functionalization with PVP-stabilized NpsAg. PVP contains pyridine groups (basic groups) and carbonyl groups (acidic groups) to the adsorbent material, so the adsorption increases from 6.83 to 9.55 mg/g. The use of stabilizing agents in the synthesis of nanoparticles is an additional source of active sites. Table 2 summarizes some of the physicochemical properties of adsorbent materials functionalized with silver nanoparticles and their adsorbent capacity from the literature. Figure 2 shows a scheme of functionalization of an adsorbent material with silver nanoparticles showing the modification of the surface area, active sites, and surface charge.

### 1.3. Silver Nanoparticles as Plasmonic Colorimetric Sensors

The presence of heavy metals in water bodies represents a threat to all living organisms due to their nonbiodegradable properties and bioaccumulation, causing high toxicity even at low concentrations [54]. The detection and removal of heavy metals in trace concentrations is a necessity in water remediation processes. Conventional techniques for detecting contaminants have high sensitivity and specificity; however, they are very sophisticated processes, with high purchasing and operating costs. In addition to requiring a long time for analysis, they are not portable, making it difficult to perform in situ analysis [55,56]. The development of highly sensitive and selective analytical tools, in addition to being simple, fast, and economical, is an environmental and health need that can be met using colorimetric methods based on metal nanoparticles.

**Figure 3:**
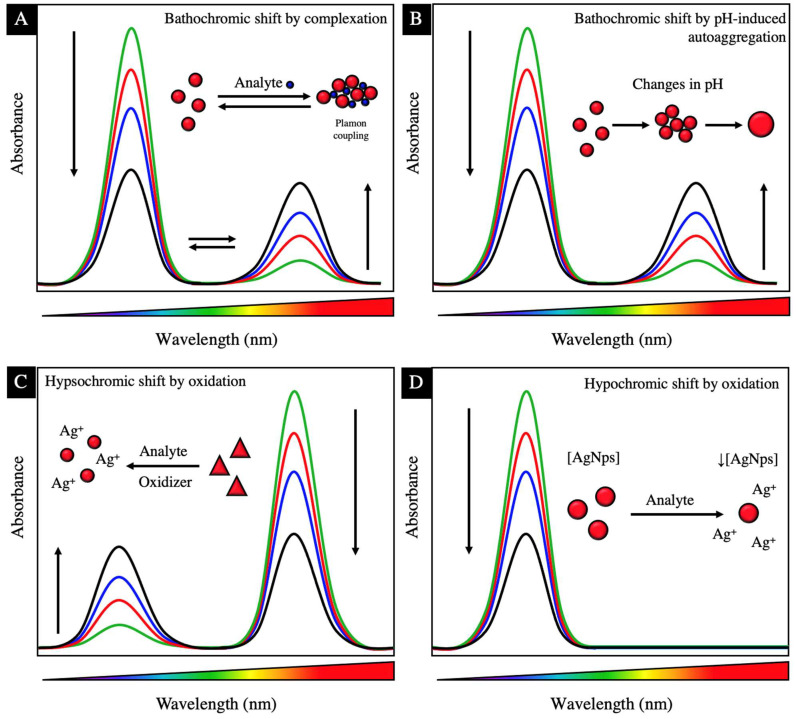
Scheme of the surface resonance plasmon shifts of silver nanoparticles by: (**A**) agglomeration; (**B**) particle growth; (**C**) change in shape; (**D**) change in concentration.

Metallic nanoparticles, when interacting with visible radiation, produce a phenomenon of surface plasmon resonance (SPR) where the conduction electrons of the nanoparticle collectively oscillate with a specific frequency, absorbing such energy. The SPR phenomenon is an optical property that allows nanoparticles to be used as colorimetric sensors in the detection of various contaminants, such as heavy metals. The basis of colorimetric methods using metallic nanoparticles is the perturbation of the SPR where variations in the size, shape, and distance between particles (aggregates) cause a displacement of SPR and therefore a change in the coloration of the solution, making perturbations visible and leading to a detectable signal [55,57,58]. Silver nanoparticles can interact with an analyte in the following three ways to trigger detection by colorimetry: (1) aggregation, (2) oxidation, and (3) change in size/morphology/concentration. Figure 3 shows a diagram of the surface resonance plasmon shifts of silver nanoparticles due to agglomeration, growth, change in shape, and change in concentration. Table 3 shows various studies where silver nanoparticles are used for the detection of heavy metals.

A decrease in the distance between nanoparticles produces aggregation of the nanoparticles, inducing a strong plasmon coupling between nearby nanoparticles, causing a bathochromic shift (toward longer wavelengths) of the SPR [57,59]. Yoosaf et al., 2007 [58] showed the detection of Pb^2+^ using NpsAg stabilized with gallic acid. The nanoparticles showed a spherical morphology, size of 32 nm with an SPR at 429 nm, and a yellow solution color that upon contact with a concentration of 5–150 μM of Pb^2+^ caused a shift of SPR to 456 and a change in color to red. Gallic acid (GA) is used as a stabilizing agent, where the carboxylic group of the molecule interacts with the silver nanoparticle through electrostatic interactions, leaving the hydroxyl groups of the ring on the surface. The interaction of Pb^2+^ and the silver nanoparticles occurs through the stabilizing agent. The phenolic hydroxyl groups of gallic acid form a multivalent coordination with the Pb^2+^ cations, causing the formation of supramolecular aggregates. The approach of the nanoparticles to each other induces coupling of its plasmon oscillation, causing bathochromic shift. GA-AgNps with a zeta potential of −45 mV have selectivity for Pb^2+^ against Fe^3+^, Cd^2+^, Cu^2+^, Hg^2+^, Ni^2+^, Zn^2+^, Mg^2+^, and Ca^2+^; however, when the zeta potential decreases, interactions with other cations are observed: −42 mV (Pb^2+^, Fe^3+^), −40 mV (Pb^2+^, Fe^3+^, Cu^2+^), indicating that the surface charge of the nanoparticles is an important factor in the complexation selectivity (Figure 3A).

The use of stabilizing agents is a determining factor in the formation of complexation with the metal, the orientation or configuration, and what functional groups remain on the surface of the nanoparticle (exposed) to participate in the complexation. The methods for detecting aggregation by complex formation are reversible using a stronger chelating agent such as ethylenediaminetetraacetic acid (EDTA), where the nanoparticle returns to its original SPR [58].

Huang et al. (2016) [60] obtained NpsAg stabilized with 1-amino-2-naphthol-4-sulfonic acid (ANS) for the detection of Cd^2+^. Through an infrared analysis, it was determined that the ANS bound to the surface of the nanoparticle through the interaction of the deprotonated sulfonic group (-SO_3_H), leaving the -NH and -OH groups on the surface exposed, which are responsible for the interaction of ANS-AgNps with Cd^2+^. The ANS-AgNps with spherical morphology and a size of 12 nm with an SPR at 390 nm, upon contact with a concentration of 1–10 μM of Cd^2+^, give an SPR with a bathochromic shift at 580 nm with a change in coloration of the solution from yellow to reddish-brown due to the aggregation of the particles. At pH < 4, the ANS-AgNPs are unstable and precipitate easily, due to the protonation of the -SO_3_H group (pKa = 1.5) causing a decrease in their action as a stabilizing agent. When the nanoparticles are not stable, they tend to aggregate, causing a displacement of SPR unrelated to the complexation with the metal, giving false positives in the detection. At pH 9.5, the greatest interaction of ANS-AgNP with Cd^2+^ occurs due to the deprotonation of the -NH_3_ group (pKa = 9.06), which allows the interaction with Cd^2+^; however, at higher pHs, the interaction decreases due to the formation of cadmium hydroxide. The type of species (protonated or deprotonated) of the functional groups of the stabilizing agent is a key factor in the complexation capacity and stability of the nanoparticles, so when performing the colorimetric analysis, the pH of the medium must be considered to prevent variations in the PSR not related to complexation.

Choudhury and Misra (2018) [61] obtained gluconate-stabilized silver nanoparticles (Gulc-AgNPs) and determined their stability at different pH. The AgNps are stabilized with the gluconate through interactions between the nanoparticle and the -COO- groups of the gluconate ion. In addition, the Gulc-AgNPs have a zeta potential of −55.2 mV, causing repulsion between the particles, keeping them stable. Colloidal particles with a zeta potential greater than ±30 mV are generally stable due to the strong electrostatic repulsion between the particles, preventing their aggregation [58]. The Gulc-AgNPs presented an SPR at 395 nm, which remains almost unchanged in a pH range of 3.67 to 10.08; however, when the pH is lower than 3.67, the nanoparticles agglomerate due to the protonation of the carboxylate group of the gluconate (pKa = 3.86), preventing the gluconate from having a stabilizing effect, which decreases the zeta potential and the nanoparticles grow and form aggregates. As the pH decreases to 6.8, 4.6, 3.9, and 2.4, the zeta potential decreases to −52, −43, −24, and −15 mV and the particle size increases to 10, 12, 22, and 67 nm, respectively. The same phenomenon is observed at a pH higher than 10.0. At pH 10.03, 10.42, and 11.06, the zeta potential decreases to −53, −31, and −2.7 mV and the particle size increases to 6.6, 21.8, and 12.7 nm. The agglomeration at a basic pH may be due to the replacement of gluconate anions on the surface of the nanoparticle by OH^−^ ions. When nanoparticle growth occurs due to the effect of pH, there is a bathochromic shift of the SPR, which is why we insist on adjusting the pH of the solution to be treated to the pH of maximum stability of the nanoparticles to be used to avoid false positives caused by pH-induced self-aggregation and not aggregation by complexation (Figure 3B).

Narayanan and Han, 2017 [62] synthesized alginate-stabilized NpsAg (Alg-NpsAg) with a spherical morphology of 10–20 nm in diameter with an SPR at 400 nm. The Alg-NpsAg were used for the detection of Mn^2+^, which upon encountering with the metal presented a color change from pale yellow to brownish yellow. In addition, a decrease in zeta potential was observed when increasing the concentration of Mn^2+^ in the media. The zeta potential of the Alg-NpsAg is −54.1 mV and by increasing the concentration of Mn^2+^ from 1–10 μM, the zeta potential gradually decreases to −43.4 mV at 10 μM of Mn^2+^, in the same way that the size of the nanoparticles gradually increases with the increase in the concentration of Mn^2+^, evidencing the agglomeration of the nanoparticles.

The detection of analytes based on the oxidation of AgNps is another frequently used method where the presence of certain analytes in solution oxidizes the nanoparticles, causing a change in their morphology. Yoon et al., 2019 [63] synthesized citrate-stabilized AgNps (Cit-AgNps), which presented a triangular morphology with a size of 40.3 nm and a zeta potential of −30.3 mV for the detection of Ni^2+^. The Cit-AgNps, having a triangular morphology, have an SPR at 335, 475, and 750 nm, which present a hypsochromic shift (toward shorter wavelengths) in contact with a Ni^2+^ solution. The band at 750 nm disappears and a band at 480–500 nm becomes more pronounced, causing a change in the coloration of the solution from blue to yellow, and TEM micrographs show a change in morphology from triangular to circular nanodisks with the addition of Ni^2+^. The Ni^2+^ ion coordinates with the Cit-AgNps through the union of the Ni^2+^ with the citrate oxygen atoms on the surfaces of the triangular AgNps, without the presence of aggregation, which suggests that the Ni^2+^ does not act as a coordination center of the metal between two nanoparticles. Due to the binding of Ni^2+^ on the surface of the nanoparticles, the zeta potential was reduced from −30 to −2.49 mV. The Cit-AgNps-Ni^2+^ union acts as a catalyst for the generation of H_2_O_2_, which acts as an oxidative etchant, where the sharp vertices of the triangular nanoparticles are oxidized for the formation of circular nanodisks and release of Ag^+^, which causes a decrease in the nanoparticle size of from 40.3 to 20 nm. The oxidizing agent preferentially attacks the facets with the highest surface energy such as {111} because they are not completely protected by the stabilizing agent, causing an inhomogeneous oxidation.

Chen et al., 2013 [56] synthesized triangular AgNps stabilized with 1-dodecanethiol (C_12_H_25_SH) and used them in the detection of Hg^2+^ with the presence of I^−^. The 1-dodecanethiol binds to the surface of the AgNp due to the strong affinity of the thiol groups with silver (S-Ag). Having the presence of Hg^2+^ in the medium, the Hg^2+^ ions extract the thiols of the vertices of the triangular nanoparticle due to the following: (1) the stability constant (logK_f_) of Hg(SCN)n (21.8) that is greater than Ag(SCN)n (10.08), where SCN is similar to -SH, for which Hg^2+^ is capable of removing ions from the surface of the nanoparticle; and (2) the {111} facets present greater surface energy compared to the {110} and {100} facets, because the facets are unprotected {111}, which allows the I^−^ to erode the AgNps at the vertices, causing a change in the morphology of the nanoparticles from triangular to circular and a change in the color of the solution from blue to purple. The etching process is facilitated by the presence of nanoparticles with anisotropy (Figure 3C).

**Table 3:** Silver nanoparticles used for the detection of heavy metals by colorimetric assay.

								Colorimetric Assay		
Ref.	AgNps	Morphology	Size (nm)	Stabilizer	Zeta Potential (mV)	SPR (nm)	Solution Color	Linear Interval	LOD	Detected Contaminant
[63]	AgNPrs	Triangular	40.3	Citrate	−30.3	335/475/750	Blue	0–30 μM	21.6 nM	Ni^2+^ pH 8
	AgNPrs-Ni^2+^	Circular nanodisks	≈20		−2.49	480–500	Yellow			
[56]	Ag NPRs	Triangular		1-dodecanethiol			Blue	10–500 nM (R 0.995)	3.3 nM	Hg^2+^ pH 5
	AgNPRs-Hg^2+^	Circular					Purple			
[64]	AgNPs	Spherical	20 ± 2	Casein peptide		410	Yellow	0.08–1.44 μM (R^2^ = 0.973)	0.16 μM	Cu^2+^
	AgNPs-Cu^2+^					520	Red			
[65]	AgNPs	Spherical	15.4 ± 3.9	Starch	−28.7 ± 1.6 (pH 6.8)	408	Yellow	0.7 to 7 mg/L	0.1 mg/L	Fe^3+^
	AgNPs-Fe^3+^					blue shift	Colorless			
[62]	AgNPs	Spherical	10–20	Alginate	−54.1	400	Pale yellow	1–10 μM (R^2^ = 0.985)		Mn^2+^
	AgNPs-Mn^2+^				−43.4	500	Brownish yellow			
[55]	AgNP-S	Spherical	30	GSH		400	Yellow	5–400 μM (R^2^ = 0.99)		Ni^2+^, Co^2+^, Cd^2+^, Pb^2+^, As^3+^
						550	Red			
	AgNP-P	Nanoplate	40	GSH			Blue			
			<40				Colorless			
	AgNP-R	Nanorod	400	CTAB/GSH		750	Pale blue			Co^2+^
			<400			300–550/750	Dark green			
[58]	AgNPs	Spherical	32	Gallic acid	−45 (pH 4.5–5.0)	429	Yellow	0–35 μM		Pb^2+^
	AgNPs-Pb^2+^	Aggregates				456	Red			
[60]	AgNPs	Spherical	12	ANS		390	Bright yellow	1.0–10 μM (R^2^ = 0.997)	87 nM	Cd^2+^ pH 9.8
	AgNPs-Cd^2+^	Aggregates	28			580	Reddish-brown			
[61]	AgNPs	Pseudo-Spherical	9.5 ± 2	Gluconate	−55.2 (pH 7.92)	395	Yellow	0.5–2.25 μM (R^2^ = 0.984)	0.2 μM	Pb^2+^
	AgNPs-Pb^2+^	Aggregates				524	Pinkish red			
[66]	AgNPs	Pseudo-Spherical	15 nm	Chitosan		398	Brownish-yellow	1–500 μM	0.53 μM	Fe^3+^
	AgNPs-Fe^3+^						Colorless			
[67]	AgNPs	Spherical		CCA		396	Yellow	0.22–3.18 μM	0.13 μM	Cd^2+^
	AgNPs-Cd^2+^	Aggregates				522	Orange			

Metal detection can also be performed by monitoring the concentration of the nanoparticles, depending on the stabilizing agent used and the morphology of the nanoparticle, which causes homogeneous oxidation in the particle. Vasileva et al., 2019 [65] synthesized AgNps stabilized with starch, which presented a zeta potential of −28.7 mV. A zeta potential below ±30 mV indicates low stability, probably caused by the type of stabilizing agent used, where the starch sterically stabilizes the nanoparticles. The addition of Fe^3+^ to the Starch-AgNps solution caused the gradual decrease in the intensity (hypochromic shift) of the SPR at 408 nm, accompanied by a slight hypsochromic shift, and the color of the solution changed from yellow to colorless caused by a redox interaction between the nanoparticles and the Fe^3+^ ion. The presence of Fe^2+^ does not produce changes in the SPR. The presence of Fe^3+^ ions on the surface of the nanoparticles produces the reduction of Fe^3+^ to Fe^2+^ and the oxidation of Ag^0^ to Ag^+^. The AgNps decompose with their oxidation and the Ag^+^ ions diffuse into the solution, which is why a decrease is observed in the intensity of the SPR as the concentration of nanoparticles decreases, and the hypsochromic shift may be due to the gradual decrease in the size of the nanoparticles (Figure 3D).

Sung et al., 2013 [55] synthesized AgNps stabilized with glutathione (GSH) of nanosphere, nanoplate, and nanorod morphology, each by a different synthesis route and observed the effect of the shape of the nanoparticle on the perturbation of the PSR for metal detection. The spherical nanoparticles had a size of 30 nm and SPR at 400 nm, which in the presence of metals, aggregation of the particles occurs due to complexation with carboxylates, resulting in a bathochromic shift of the SPR at 550 nm with the change in the color of the solution from yellow to red. The selectivity of GSH-AgNps with spherical morphology is poor but can be applied with a universal colorimetric sensor for various metal ions. On the other hand, the nanoplate nanoparticles (triangular) have a size of 40 nm and three SPR bands that when in contact with various metal ions, the SPR bands disappear and the color of the solution changes from blue to transparent. Through a TEM analysis, a considerable decrease in the number of nanoparticles and a size smaller than the initial one of >40 nm is observed, due to the oxidation of the AgNps and the release of the Ag^+^ ions, which ends up completely ionizing the nanoparticles. The roller-shaped nanoparticles presented a length of 400 nm, an SPR located at 750 nm, and a detection for Co^2+^ where the color of the solution changed from pale blue to dark green with a decrease in the intensity of the SPR at 750 nm and the appearance of a new one at 330–550 nm, which indicates the formation of smaller particles in length < 400 nm. The morphology of the particle is an important factor in the detection mechanism where spherical particles tend to agglomerate. Anisotropic particles such as triangular particles and rollers induce oxidation of the most energetic and exposed facet {111}.

### 1.4. Silver Nanoparticles as Signal Amplifiers in Infrared and Raman Spectroscopies

Infrared and Raman spectroscopies are two analysis techniques that make use of light, either in the infrared or visible range of the electromagnetic spectra, to determine the chemical composition of a large variety of substances. When combined with the phenomenon called surface plasmon resonance (SPR) where the electrons of a metal oscillate collectively when exposed to light, both techniques become more sensible at detecting small amounts of an analyte due to its optical absorption being increased [68]. Since the implementation of surface enhanced spectroscopy, for the Raman (SERS) or infrared (SEIRAS) techniques, nanostructured materials composed of noble metals have been used for rough surfaces and metal nanoparticles [69]. Metal nanoparticles can function as emitters of localized surface plasmon resonance and they have been a crucial part of the design of plasmonic enhancement substrates. Among the metals used, silver and gold have had the most success in enhancing spectroscopic signals; however, silver is widely used due to it being excitable in the near-infrared and visible ranges, economically accessible, and more abundant than gold [70].

Different approaches have been studied over the years for the use of silver nanoparticles in SEIRAS and SERS, modifying characteristics such as particle morphology, spacing between particles, and using a variety of base materials. For example, one of the most used techniques for nanoparticle fabrication is chemical reduction. Usually, the metallic salt is mixed with a variety of compounds to reduce ionic silver (Ag^+^) into metallic silver (Ag^0^) such as ascorbic acid [71], gallic acid [7], trisodium citrate [72], and even ultraviolet light [73]. Along with reducing agents, compounds such as polymers are used to protect the particles from both growing and tarnishing. Other techniques include, for example, crystallization, electron beam lithography, plasma deposition, electroless deposition, and electro-deposition, among others. The particle fabrication method is strongly related to the morphology obtained. For example, chemical reduction often produces spherical particles; however, the addition of chelating agents and metal–organic frameworks allows for complex structures to be formed on the nanometric scale. Techniques such as plasma deposition allows to produce polymeric sacrificial templates that end up being substituted for metal nanostructures. This is because complex morphologies permit the formation of hot spots, or chemical or electromagnetic bonds between the substrate and the analyte. It has been reported that morphologies with multiple projections or angular surfaces are more effective at amplifying spectroscopic signals [74].

**Figure 4:**
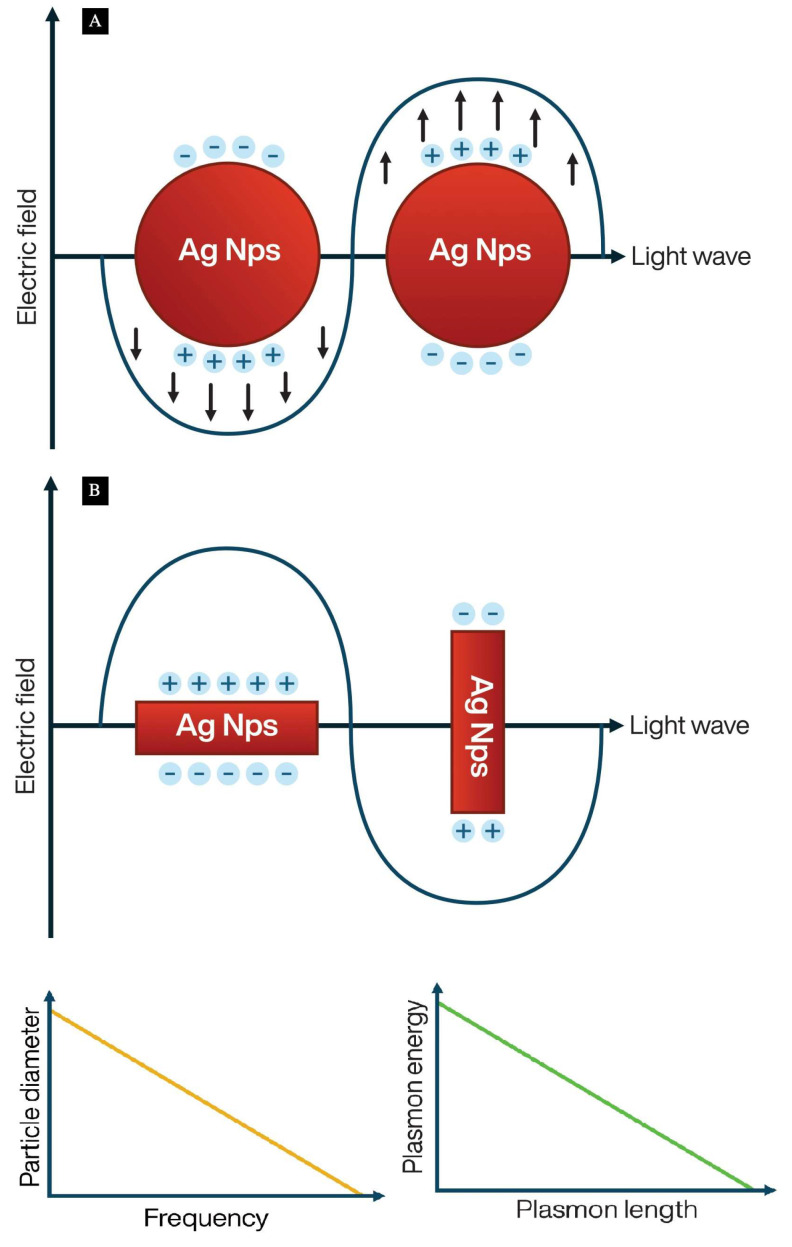
Plasmon resonance is produced when light is incided onto a rough surface or nanoparticle. The alternating light wave excites electrons on the metal, generating an oscillating dipole. The frequency of the plasmon is inversely related to the particle size. In addition, the plasmon length is inversely related to the plasmon energy. Behavior of surface plasmon resonance on isotropic particles (**A**) and anisotropic particles (**B**).

Particle morphology and size also has an influence over characteristics of the plasmon such as the resonance frequency. Because electrons move from one extreme of the particle to the other, different lengths allow for different speeds in oscillation [75]. When the frequency of incident light oscillations coincides with the intrinsic frequency of conduction electrons in the particle surface, the resonance light absorption and scattering are observed (SPR). For spherical particles, the interaction with the incident optical wave is equivalent in all directions because it is an isotropic morphology, so they present a single SPR band in the spectrum (Figure 4A). For nonspherical particles, the different orientations with respect to the incident optical wave are nonequivalent. Thus, for cylindrical particles, the surface plasmon resonance frequencies of dipole oscillations induced along and across the cylinder axis are different, giving two SPR bands in the spectrum [11] (Figure 4B). Thus, the spectra signal bands are correlated with symmetry of the particle shape. An important component of spectroscopic enhancement substrates is the base material. Usually, an enhancement substrate is formed by a dielectric material, and the rough metal surface on top. A variety of materials are used such as polymers ranging from cellulose [76], paper [77], and reclaimed compact discs [78], to SiO_2_ glass [79], among others. However, some spectroscopic enhancements could also be made using colloidal solutions of nanoparticles.

Table 4 shows all the different characteristics of the substrate that contribute to the enhancement; however, particle size and morphology are shown to be the principal key factor event when the enhancement is provided by just particles in a colloidal solution. For, example Messina et al. produced nanoparticles via reduction, using AgNO_3_, NaBH_4_, and trisodium citrate. Initially, they created small particles, which they later grew up using a solution of hydrazine and trisodium citrate to contain the particles. A solution of silver nitrate was added drop wise to increase the size of the particles. They obtained sizes from approximately 40 to 110 nm. They tested the enhancement capacity of the particles with SERS against a gold flat surface using a solution of methylene blue 10^−4^ M. Although the Raman spectra with silver nanoparticles shows significant enhancement in comparison with gold, they did not provide the enhancement factor of the material, nor did they test the enhancement properties of the Ag nanoparticles against the same morphology with gold, which could potentially make a huge difference.

Zhao et al. (2015) [80] produced AgVO_3_ nanoribbons decorated with silver nanoparticles. The particles were obtained by chemical reduction with AgNO_3_ and NaBH_4_ as precursors, and, although the size of the particles is not specified, SEM micrographs show that they are around 100 nm and less. The material is proposed for photocatalytic applications; however, the vanadate ribbons were analyzed in Raman spectroscopy, with and without silver nanoparticles, showing an increased improvement in the signal when the nanoparticles are present. Blanco-Formoso et al. [81] obtained magnetic silver nanoparticles using iron ions as the magnetic component. Initially, ascorbic acid was dissolved in boiling water, then, a solution of silver and iron nitrate was added to the ascorbic acid solution at different concentrations. The morphology of the particles was spherical, and the diameters ranged from 13 to 34 nm. Although the enhancement factor was not shown, they were able to amplify a concentration in the micromolar range.

Eid et al. [82] prepared silver nanoparticles using a hot solution of silver nitrate to which a trisodium citrate solution was added. The particle solution was deposited onto electropolished aluminum sheets a left to dry at 100 °C. Reyes Gómez et al. [74] produced solutions of silver nanoparticles with different morphologies, such as star-like morphology using silver nitrate as the metallic precursor and hydroxylamine as well as sodium citrate as reducing agents and silver nanospheres using silver nitrate and hydroxylamine as the reducing agent. Silver nanoplates with a triangular shape were obtained using silver nitrate, sodium citrate, and potassium bromide. Unfortunately, no enhancement tests were carried out, or an enhancement factor calculated.

Scuderi et al. [83] proposed an array of disk-like nanoparticles using electron beam lithography. The substrates were prepared with a glass base, and chromium or ITO as an anchor and conductive layer. The diameter of the disk varied from 50 to 190 nm, and the thickness of the disks was 40 nm. Although the disks are proposed for plasmonic applications, the disk were not tested for enhancing of the spectroscopic signals. Jiang et al. [84] produced an enhancement substrate using a metal–organic framework and growing in situ silver nanoparticles using AgNO_3_ and tannic acid as a precursor and reducing agent, respectively. The particle size was estimated as 40 nm. The overall shape of the framework was an icosahedron, allowing for a high densification of particles and increased surface area. The limit of detection achieved was 0.32 pM.

Díaz-Liñán et al. [85] obtained flower-like particles using silver nitrate and sodium citrate tribasic. In this instance, the substrate was prepared using paper that was covered in nylon-6. The nitrate solution was dropped onto the surface of the nylon layer and irradiated with 254 nm UV light for the reduction to silver. The substrates were tested for the determination of ketoprofen. An enhancement factor of 1.97 × 10^4^ was achieved for SERS. The use of nylon prevented the analyte from being absorbed into the paper. Riswana Barveen et al. [86] made silver nanoparticles with flower-like morphology, using silver nitrate as the precursor and trisodium citrate, PVP, and ascorbic acid. They used ice to lower the temperature of the reaction and mixed three solutions of silver nitrate, PVP, and trisodium citrate. A solution of ascorbic acid was added drop wise to reduce the silver. In this instance, the morphology of the particles was that of flower; however, they induced the growth of particles onto the flowers by adding silver nitrate and trisodium citrate to the nanoflower solution. The average diameter of the flowers was 470 nm. No diameter was provided for the in situ grown nanoparticles. Particles were tested for the SERS technique with a maximum enhancement factor of 10^7^ for Congo red, and 10^12^ for Rhodamine 6G.

Guo et al. [87] produced core/shell nanoparticles using gold and silver. The morphology of the particles was that of a gold sphere encased in a silver cube. Initially, the gold nanoparticles were synthesized using chloroauric acid, which was reduced in an aqueous solution and stabilized using CTAB. The resulting nanoparticles were grown from 3 nm to 6 nm using a solution of chloroauric acid, ascorbic acid, and CTAC. To encase the gold nanoparticles in silver, a solution of silver nitrate, ascorbic acid, and CTAC was used. Different sizes of cubic core/sell nanoparticles were obtained, ranging from approximately 15 to 43 nm. Ambroziak et al. [88] prepared cubic nanoparticles using ethylene glycol, sodium sulfide, silver nitrate, and PVP. The ethylene glycol was heated to 170 °C then the sodium sulfide and PVP were added to the solution. Finally, the silver nitrate was introduced. The reduction of silver cations went on until the solution turned green. The cubic silver nanoparticles were deposited on different types of substrates such as titanium, TiO_2_, TiO_2_ nanotubes, and silicon. The enhancement factor for each individual type of substrate was calculated as 2.8 × 10^5^ for Ti, 9.1 × 10^4^ for TiO_2_, 3.7 × 10^6^ TiO_2_ nanotubes, and 2.6 × 10^5^ for Si, with the TiO_2_ nanotubes being the most successful at amplifying the spectroscopic signals because the complex morphology of the substrate allows for a greater number of hotspots.

Li et al. [89] developed rod-like silver nanoparticles where a gold nanoparticle was used both as a seed and a directing agent. The gold nanoparticles were synthesized in different stages, first by reduction using chloroauric acid as the gold precursor and sodium borohydride as well as trisodium citrate to obtain the seeds. The seeds were added into a growth solution made of CTAB, chloroauric acid, silver nitrate, hydrochloric acid, and ascorbic acid. This method produced bipyramidal nanoparticles with diameters in the range of 30 to 70 nm. These bipyramids were introduced into a silver nitrate and CTAC solution with grown silver nanorods of different lengths ranging from 270 to 1300 nm, where each length had a varying optical behavior. The silver nanorods were coated with silica using a solution of TEOS, NaOH, and CTAB to improve silver chemical stability. The authors calculated the enhancement factor of the CTAB that was used in the synthesis to be 1 × 10^3^; however, they claim that the enhancement is lower than previously reported because the probe molecule is encased inside the silica layer.

**Table 4:** Different characteristics of the substrate for enhancement of a signal in infrared and Raman spectroscopy.

Ref.	Technique	Particle Obtention	Base Material	Morphology	Particle Size (nm)	Max. Enhancement Factor
[90]	SERS	Chemical reduction	Colloidal solution	Spheres	39–100	
[80]	SERS	Chemical reduction	Silver vanadate	Spheres	25–50	
[81]	SERS	Chemical reduction	Colloidal solution	Spheres	13–37	
[82]	SEIRAS	Chemical reduction	Aluminum	Spheres	10–60	×2
[74]	SEIRAS/SERS	Chemical reduction	Colloidal solution	Stars, spheres, triangular plates	110, 50, 38	
[83]	SEIRAS/SERS	Electron beam lithography	Glass/Chromium/ITO	Disks	50–190	
[84]	SERS	Chemical reduction	MOF MIL-101	Icosahedron	40	1.8 × 10^5^
[85]	SERS	Photoreduction	Paper/Nylon 6	Flowers		1.97 × 10^4^
[86]	SERS	Chemical reduction	Colloidal solution	Flowers/Particles	470	1 × 10^12^
[87]	SERS	Chemical reduction on gold	Colloidal solution	Cubes	47 × 21	272.15 × 10^6^
[88]	SERS	Chemical reduction	TiO_2_ nanotubes	Cubes	45	3.7 × 10^6^
[89]	SEIRAS	Chemical reduction/crystallization	Colloidal solution	Rods	30–70	1 × 10^3^
[91]	SERS	Electroless deposition	Anodized aluminum oxide	Tentacles	100–200	1.2 × 10^7^

Wang et al. [91] used an anodized aluminum oxide template, a substrate with the morphology of “nanotentacles”, in the words of the authors, to resemble the surface of a gecko’s feet, which was fashioned out of PDMS and later decorated with gold–silver nanoparticles. Initially, the polymeric substrate was covered in a colloid of gold particles, and later by electroless deposition, silver was grown on the surface of the gold particles, achieving particles with a flower-like morphology. Although the size of the nanoparticles was not specified, SEM micrography showed it was in the range between 25 and 50 nm. Garibay-Alvarado et al. [92] produced a flexible ceramic membrane using ZrO_2_ and Mullite. The ceramic precursors were tetraethyl orthosilicate, aluminum nitrate nonahydrated, and zriconium butoxide. The silver nanoparticles were obtained by chemical reduction of a silver nitrate solution and added to the fibers by immersion. An enhancement factor of 1.5 × 10^6^ times was achieved with a concentration of 1 nM pyridine.

### 1.5. Silver Nanoparticles as Plasmonic Photocatalysts

Photocatalysis is considered one of the best methods for environmental purification because additional chemical compounds such as strong oxidants like ozone, hydrogen peroxide, and chlorine are not introduced into the system. In addition, energy consumption is also much lower than in the case of advanced oxidation technologies [93]. Photocatalysis is a photochemical reaction that converts solar energy or photons into chemical energy on the surface of a catalyst. In the photocatalysis reaction, the light falls on the surface of a semiconductor, where the electrons absorb it and become excited, generating pairs of electrons. These pairs of electrons can lead to the generation of free radicals such as hydroxyl or reactive oxygen species that produces secondary reactions, which allow rapid nondiscriminatory oxidation of any organic substance [94].

In photocatalysis processes, nanomaterials excited by light of appropriate wavelength generate active species that oxidize organic compounds dissolved in water; however, most of them currently have low percentages of catalysis, adsorption capacity, limitations in their photocatalytic efficiency, light absorption, and high production costs. Hence the current interest of researchers in improving photocatalytic performance, either by changing electronic structural properties such as charge transfer, bandgap, morphology, and particle size, improving doping of metals and nonmetals, thereby seeking advanced photocatalytic nanomaterials. The photocatalytic activity of a solid material is the property induced by the irradiation of photons with energy equal to or greater than the energy of the band gap of the material on its surface, which causes the e^−^ of the band of valence (VB) to be excited toward the conduction band (CB) and leave gaps in the first. In this way, hole electron (e^−^-h^+^) pairs called excitons are generated, which can subsequently be used to carry out redox reactions.

Photocatalysis has had great interest in applications in self-cleaning, antimicrobial coatings, and in the elimination of organic contaminants from aquatic environments, because good photocatalysis can completely degrade organic contaminants. This process does not generate toxic byproducts, which is an advantage compared to other removal methods. Currently, photocatalysis is an ecological technology for the complete degradation of dangerous organic chemicals in water such as nitrophenols, drugs, and dyes, among others [93,94,95].

Titanium oxide or titania (TiO_2_) is one of the most used semiconductors photocatalysts, because it has many advantages such as good stability, strong redox capacity, nontoxicity, low-cost synthesis processes, and high availability. But it presents a disadvantage in the inability to absorb visible light, due to its wide bandgap of approximately 3.0 eV for rutile and 3.2 eV for anatase, limiting its application with solar radiation. Hence the search to improve the photocatalytic performance of titania by modifying its surface, doping, and preparing composite nanostructures [96,97,98].

Modification with metal nanoparticles has been one of the most used methods both to improve the activity under ultraviolet light and activation of titania toward visible light irradiation. Under UV irradiation, metal nanoparticles function as an electron reserve that inhibits the recombination of charge carriers. While under visible light irradiation, either due to the narrowing of the bandgap or due to the energy/electron transfer of the modified titania, it causes a visible response [93,94,95,96,97,98,99,100]. Recently, there are reports of modification of titania with noble metal NPs that absorb visible light due to their localized surface plasmon resonance. These have been termed “plasmonic photocatalysts”. Due to their photocatalytic properties resulting in very wide irradiation ranges, the modified materials are very promising for environmental and energy conversion applications [93,100,101].

With the development of nanotechnology, new applications of silver nanoparticles have increased successfully in wastewater treatment due to their specific characteristics, such as greater surface area, smaller size, better distribution, and designed morphology. These specific intrinsic properties give nanoparticles candidate structures for their application in catalytic degradation [102]. Silver nanoparticles are well known for their antibacterial activity, chemical stability, conductivity, and catalytic activity properties that make it the most preferred noble metal for the synthesis of stable photocatalytic nanoparticles capable of degrading organic compounds [103,104,105].

**Figure 5:**
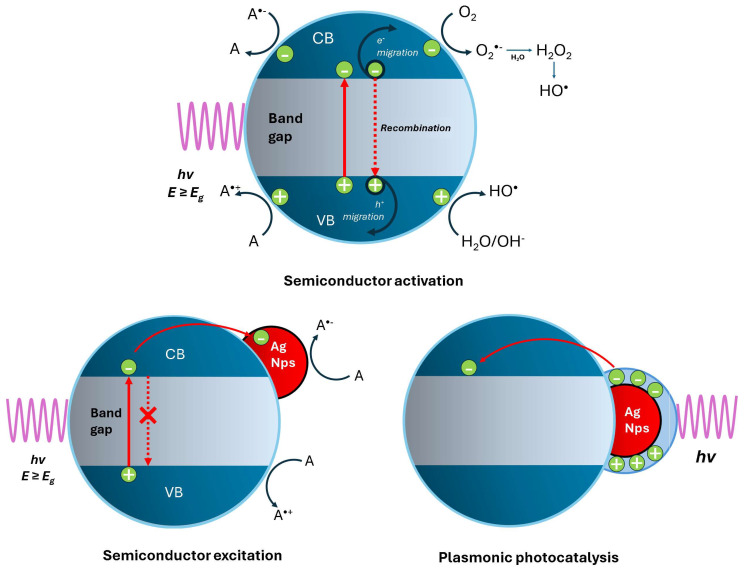
Scheme for the activation of a semiconductor, excitation of a semiconductor, and plasmonic photocatalysis.

The mechanism of photocatalysis of a semiconductor is shown in Figure 5 and consists first of the excitation of the semiconductor with light of energy equal to or greater than its bandgap energy, resulting in the formation of pairs of charge carriers, the electrons and holes (e^−^/h^+^). The second step is the migration of charge carriers to the semiconductor surface (recombination) and the reaction of charge carriers with adsorbed species (recombination). The h^+^ generated in the valence band are strong oxidants and the e^−^ in the conduction band act as reducers. The generation of these pairs can bring with it different effects. One is that they can recombine and release their energy in the form of electromagnetic radiation or heat. They can also migrate to the surface of semiconductors and react with adsorbed molecules. In the presence of water and oxygen molecules in the system, reactive oxygen species (ROS), such as superoxide (•O^2−^), hydrogen peroxide (H_2_O_2_), and hydroxyl radicals (•OH), are formed and can easily react with all organic and inorganic compounds. Hydroxyl radicals are known as the strongest oxidants and are safe in aqueous processes. Noble metals in the form of adsorbed complexes and metal deposits have been extensively investigated to improve the photocatalytic activity of photocatalysts such as titania under UV irradiation, and the improvements in its photocatalytic activity originate mainly in the prolongation of the useful life of the charge carriers (electrons and photogenerated holes), because noble metals serve as electron sinks; therefore, accelerating the transfer of electrons from the semiconductors to substrates as shown in Figure 5. The three main mechanisms for plasmonic photocatalysis under visible light irradiation are energy transfer, charge (electron) transfer, and plasmonic heating [93,98].

The catalytic activities of nanoparticles with tetrahedral, cubic, and spherical shapes indicate that the largest surface atoms at the edges and corners give rise to the highest catalytic activity. Crystal planes play an important role in catalysis. The literature mentions differentiation between ultrasmall clusters at 1 nm or less in diameter, colloids between 5 and 100 nm, and clusters in the range of 1 to 5 nm. Below 5 nm, nanoparticles have a higher proportion of faces, edges, corners, microdefects, and unsaturated bonds. Furthermore, the electronic properties of ultrasmall nanoparticles could be significantly different, making studies in this area of research quite active and promising because they allow the development of catalysts based on nanoparticles with specificity and adjustable efficiency. The design of efficient water treatment systems based on photocatalytic processes with nanoparticles requires an adequate analysis of the metal properties in relation to the purpose and application conditions [106].

## 2. Limitations of the Use of Silver Nanoparticles in Water Remediation

Silver nanoparticles have a potential use in the remediation of water pollution, covering aspects such as the detection, removal, and/or elimination of contaminants and microorganisms; however, one point to consider are the repercussions that the use of silver nanoparticles may have without biosafety. The toxic effects of releasing silver nanoparticles into the environment have been reported in different reviews [107,108]. Technologies for water remediation must present low or no secondary contamination; therefore, the use of silver nanoparticles as a tool in environmental water remediation must be limited or conditioned by the use of an innocuous support matrix (solid substrate) with high affinity to AgNps (e.g., hydroxyapatite) [12,109] to avoid their release into the environment or if the application or treatment involves the use of nanoparticles in suspension, it is recommended to use catchment traps of the same support material. The immobilization of AgNps on a substrate is a requirement for its applications at an industrial level as well as in situ, allowing its use safely and avoiding damage to the environment due to its release. Furthermore, the use of nanotechnology as a tool in water remediation requires an exhaustive review and compression of the structure–physicochemical properties–activity relationships to ensure the reliability of the process.

## 3. Conclusions

The use of silver nanoparticles is a viable alternative in the optimization of environmental remediation processes; however, according to the application, silver nanoparticles must possess certain properties to improve their performance. For antibacterial activity, the use of silver nanoparticles with a size less than 10 nm, morphology with a high percentage of reactive facets {111}, and a positive surface charge is suggested. These parameters will improve the interaction of the nanoparticles with the bacterial cell and induce a greater antibacterial effect. Therefore, the type of morphology of the nanoparticle is a key factor in its reactivity and interaction contact with the bacteria. In silver nanoparticles used as a surface modifier in adsorbent materials, it is recommended to find the optimal functionalization concentration where the textural properties are increased and according to the type of adsorbate used to adsorb; the surface charge and the number of active sites can be modified by using a stabilizing agent, which will promote greater adsorbent capacity. For the use of silver nanoparticles as colorimetric sensors, the use of a stabilizing agent selective to the type of analyte to be detected is recommended to induce a selective complexation. The nanoparticles must have a surface charge greater than ±30 mV and the pH of the solution must be adjusted to the pH of maximum stability of the nanoparticles to avoid self-aggregation due to destabilization or low selectivity. Spectroscopic enhancement substrates with silver nanoparticles have a variety of advantages. They can respond to the visible near-infrared range of the electromagnetic spectrum, being especially useful for infrared and Raman spectroscopy. Unlike gold, silver is an economically accessible option, enhancement factors achieved with substrates that use silver nanoparticles are considered some of the best, and the methodology for obtainment of silver nanoparticles of different shapes and sizes is very well established. Although the use of silver for enhancement has its drawbacks, such as tarnishing, much research has been made into how to protect silver nanoparticles from the environment, making silver nanoparticles a great material for spectroscopic signal enhancement. Because many conventional photocatalysts have low efficiency, little stability, and durability, the design of new photocatalysts is needed to overcome these restrictions. Through new nanometric synthesis methods, photocatalytic nanomaterials can be obtained and designed, overcoming existing restrictions; therefore, understanding the design and optimization of the properties of a photocatalytic material to achieve maximum efficiency should be considered an essential step for applications. Finally, it is concluded that silver nanoparticles have potential use as a tool in water remediation, where the selecting appropriate physicochemical properties for each application, their performance, and efficiency are improved.

## Data Availability

Not applicable.
